# Supplementary material for: Occlusive membranes for guided regeneration of inflamed tissue defects
Source: Nat Commun. 2023 Nov 24;14:7687. doi: 10.1038/s41467-023-43428-3 (PMC10673922; doi:10.1038/s41467-023-43428-3)
Supplement: Supplementary file 1 — Supplementary Information [file 41467_2023_43428_MOESM1_ESM.pdf]

Occlusive Membranes for Guided Regeneration of Inflamed Tissue Defects

Woojin Choi[1,†], Utkarsh Mangal[2,†], Jin-Young Park[3], Ji-Yeong Kim[2], Taesuk Jun[1], Ju Won Jung[4], Moonhyun Choi[1], Sungwon Jung[1], Milae Lee[1], Ji-Yeong Na[3], Du Yeol Ryu[1], Jin Man Kim[4], Jae-Sung Kwon[5], Won-Gun Koh[1], Sangmin Lee[6], Patrick T. J. Hwang[7], Kee-Joon Lee[2], Ui-Won Jung[3], Jae-Kook Cha[3,*], Sung-Hwan Choi[2,*], and Jinkee Hong[1,*]

Supplementary Note. Classification of the periodontally-related indicator species

The Socransky classification is employed to categorize periopathogens, which are bacteria associated with periodontal diseases.[1, 2] This classification scheme, based on the pioneering work of Socransky et. al using checkerboard DNA-DNA hybridization, aids in understanding the complexity of the oral microbiome and its impact on oral health.[3] The classification groups periopathogens into six complexes (clusters) based on their association with periodontal disease severity:

- Orange Complex (initial stages)

  Fusobacterium nucleatum subspecies, *Prevotella intermedia*, *Prevotella nigrescens*, *Peptostreptoeoccus micros*, *Campylobacter rectus*, *Campvlobacter showae*, *Campylobacter gracilis*, *Eubacterium nodatum*, and *Streptococcus constellatus*

- Green Complex (early to moderate periodontitis)

  Capnocytophaga species, *Campylobacter concisus*, *Eikenella corrodens,* and *Actinobacillus actinomycetetncomitans*

- Yellow Complex (moderate disease)

  Streptococcus mitis, *Streptococcus sanguis* and *Streptococcus oralis*

- Purple Complex (advanced periodontitis)

  Veillonella parvula and *Actinotnyces odontolyticus*

- Red Complex (severe disease)

  Porphyromonas gingivalis, *Bacteroides forsythus* and *Treponema denticola*

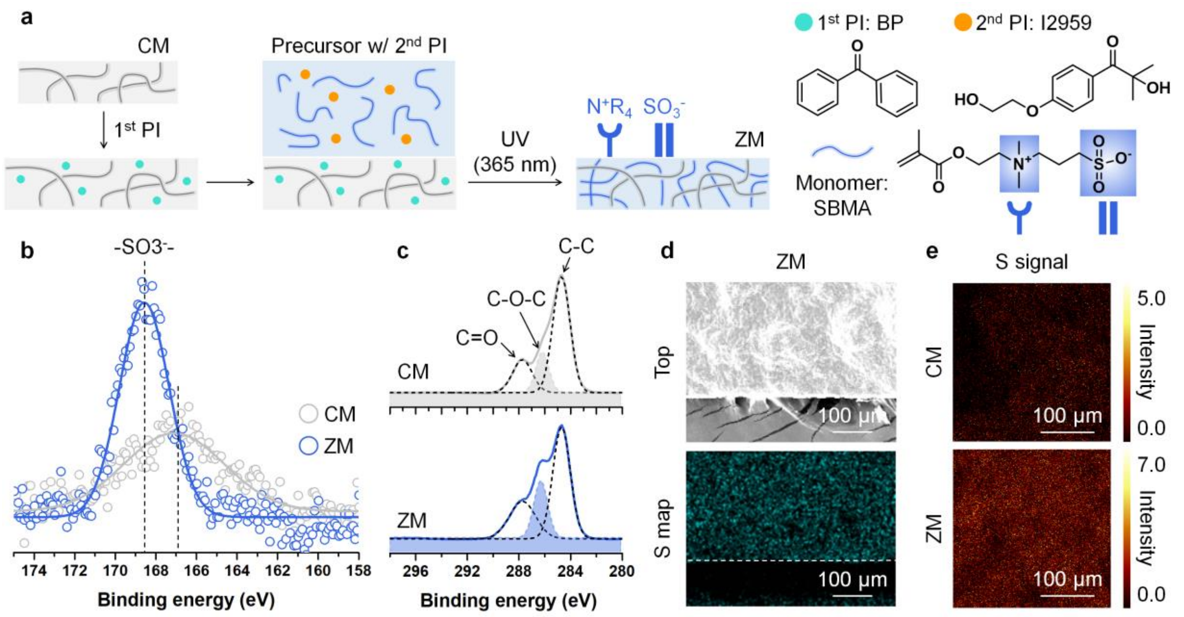

Supplementary Fig. 1. Characterization of the zwitterionic membrane (ZM). a Schematic of dual radical-based ZM preparation from the collagen membrane (CM). [4] Two photo-initiators (PI) are employed: benzophenone (BP) and 2-hydroxy-4′-(2-hydroxyethoxy)-2-methylpropiophenone (I2959). The sulfobetaine zwitterionic monomer is [2-(methacryloyloxy)ethyl]dimethyl-(3-sulfopropyl)ammonium hydroxide (SBMA). **b-c** X-ray photoelectron spectra of CM and ZM represent that zwitteration was sufficiently conducted. X-ray photoelectron spectroscopy (K-ALPHA, Thermo Fisher Scientific, USA) was utilized. **b** The distinct peak at 168.6 eV indicates (-SO^3-) of SBMA. The weak and broad peak at 167.1 eV originates from the cysteine of CM. **c** C-O-C signal at 286.3 eV is increased 1.29 times in ZM compared to CM. **d** Top view image and sulfur ion mapping result of the ZM (n=5). **e** The sulfur signals (-S-) of CM and ZM were observed by time-of-flight secondary ion mass spectroscopy (TOF SIMS 5, IONTOF, Germany). The signal intensity is enhanced 3.86 times in the ZM. Source data are provided as a Source Data file.

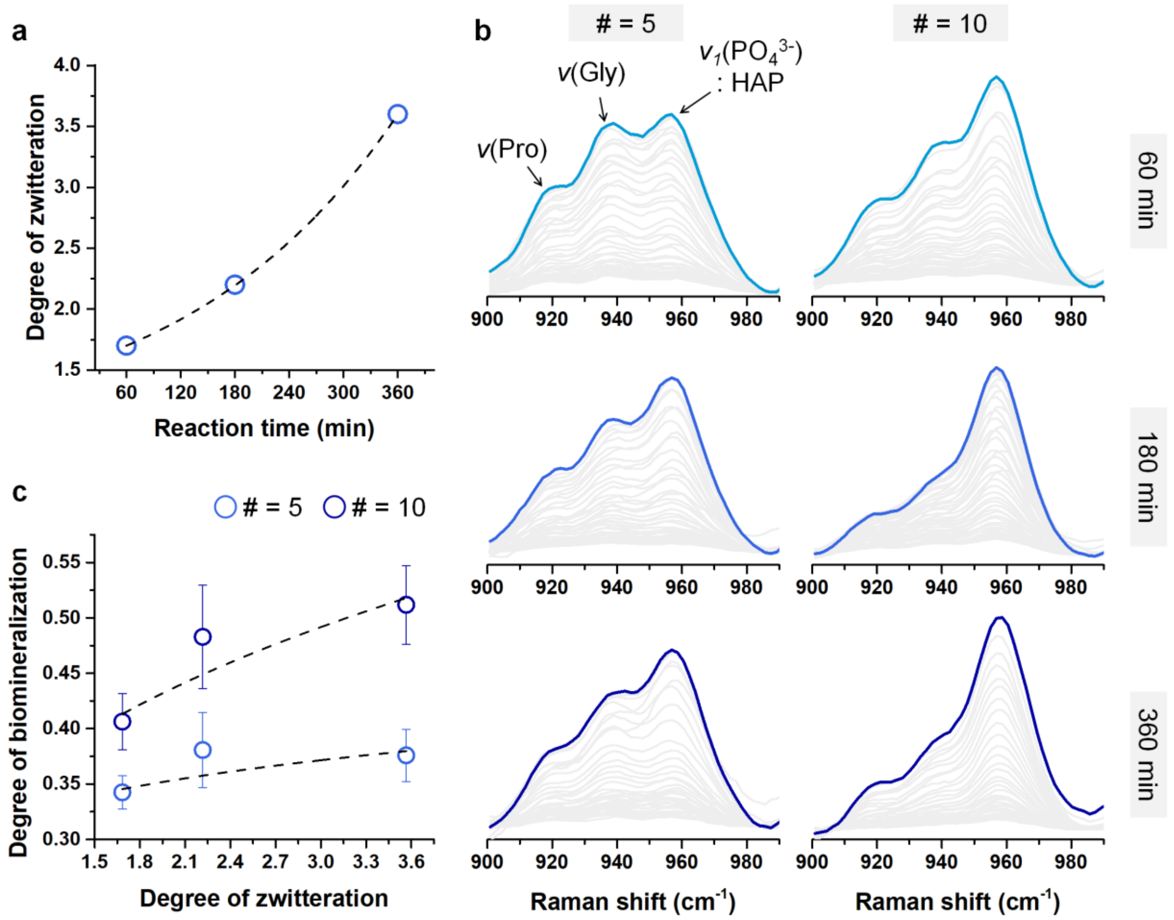

Supplementary Fig. 2. Comprehension of the biomineralization kinetics as a function of zwitteration. a The longer reaction time results in a higher degree of zwitteration. The degree of zwitteration is determined from the ($-SO^{3}-$) signal intensity at 168.6 eV of X-ray photoelectron spectra. **b** Raman depth profiles with different reaction times and numbers of PO_4^{3-}-Ca^{2+} exposure sets (#).[5] The color lines are acquired from the top surface. The light gray lines represent the depth profile up to 400 μm per 10 μm. HAP means hydroxyapatite. **c** The relationship between the degree of biomineralization and degree of zwitteration. The zwitteration facilitated biomineralization.[6] The degree of biomineralization is determined from Raman depth profiles by calculating v_1(HAP)/[v(Pro)+v(Gly)+ v_1(HAP)] value of each depth point. Source data are provided as a Source Data file.

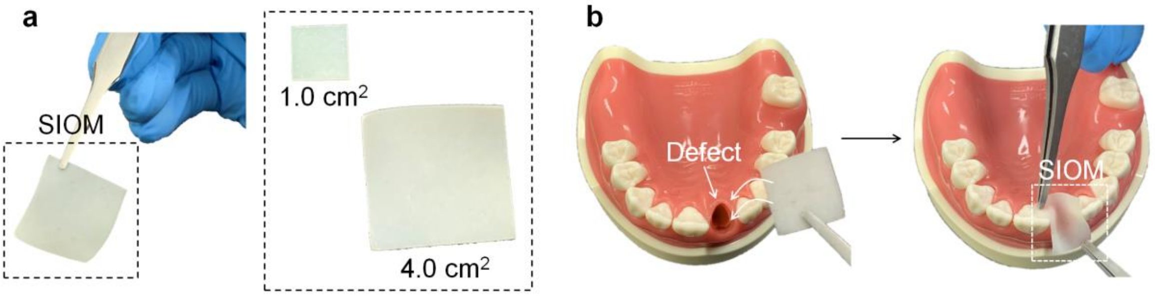

Supplementary Fig. 3. Photographs of the symbiotically integrating occlusive membrane (SIOM). a Different size of the SIOM. **b** The SIOM is tractable, enabling readily application during dental surgeries.

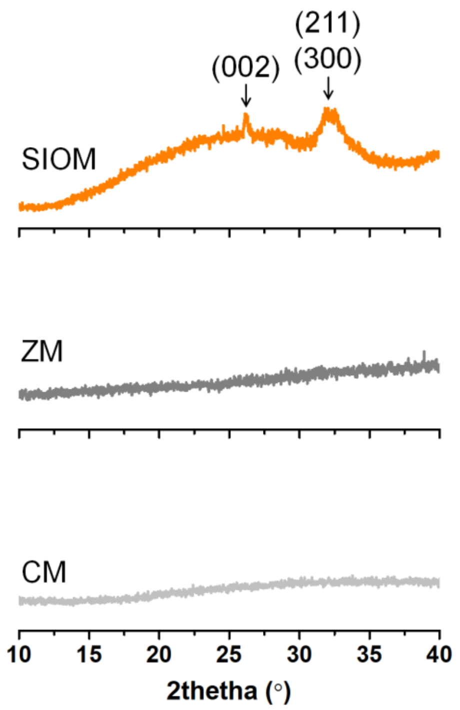

Supplementary Fig. 4. X-ray diffraction patterns of CM, ZM, and SIOM. The SIOM presents HAP-associated signals: (002) peak at 26.2°, (211) peak at 31.8°, and (300) peak at 32.5°. A high-resolution X-ray diffractometer (SmartLab, Japan) with a 9 kW X-ray source was used. Source data are provided as a Source Data file.

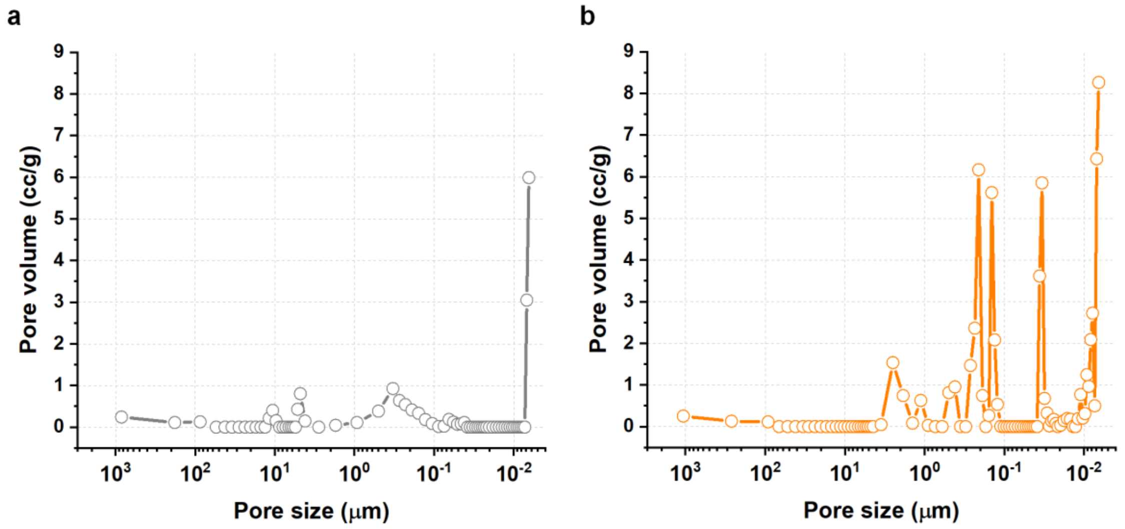

Supplementary Fig. 5. Pore size distribution profiles of **a** CM and **b** SIOM. The mercury intrusion porosimeter (PM33GT, Quantachrome) experiments were conducted. Source data are provided as a Source Data file.

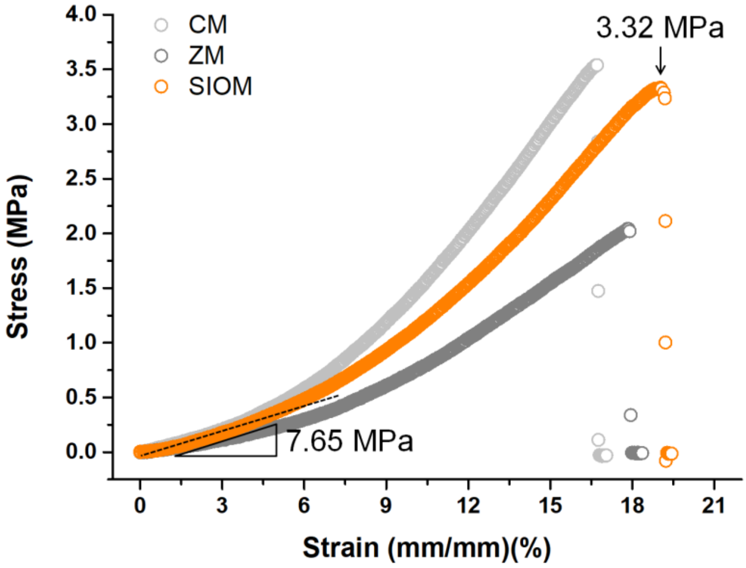

Supplementary Fig. 6. Stress *vs.* strain curves of CM, ZM, and SIOM. If the free radicals are captured by the charged side group of Arg or Lys, the peptide bond could be dissociated.[7] Due to the harsh radical condition, the mechanical property is reduced in the ZM compared to the CM. Because the strong HAP is outstanding in energy dissipation,[8] the introduction of HAP leads to a significant improvement in mechanical properties. For instance, when HAP was introduced to the acrylamide hydrogel, it enhanced the ultimate compression stress up to 2 times and ultimate tensile stress up to 3 times.[5, 9] In terms of the SIOM, the biomineralized HAP compensates for the decreased mechanical property of the ZM. Remarkably, the SIOM shows the comparable property with clinical grade CM. In detail, the ultimate stress and Young's modulus of the SIOM are 3.32 MPa and 7.65 MPa, respectively. A uniform tensile deformation of 5.0 mm min^{-1} was applied to the beam-type samples (4.0 cm^2) through a universal testing machine (Model 3366, Instron, USA).[10] Source data are provided as a Source Data file.

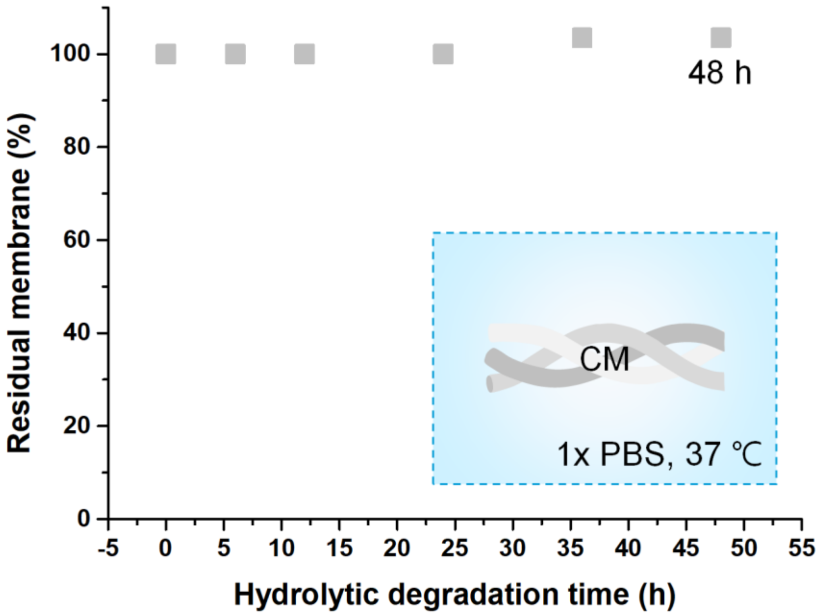

Supplementary Fig. 7. Hydrolytic degradation profile of the CM. The CM presents superior durability when it is incubated in 1× PBS buffer for 48 h at 37 °C. Source data are provided as a Source Data file.

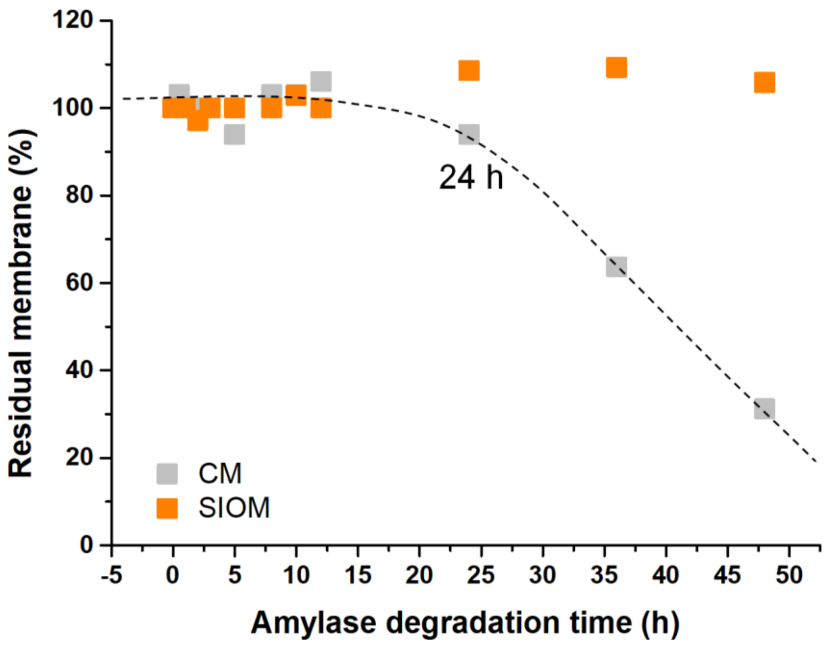

Supplementary Fig. 8. Amylase degradation profiles of the CM and SIOM. The CM and SIOM are incubated in 1,000 U/mL α-amylase solution for 48 h at 37 °C. The onset point of CM degradation is determined as 24 h. In contrast, the SIOM presents excellent enzymatic stability. Source data are provided as a Source Data file.

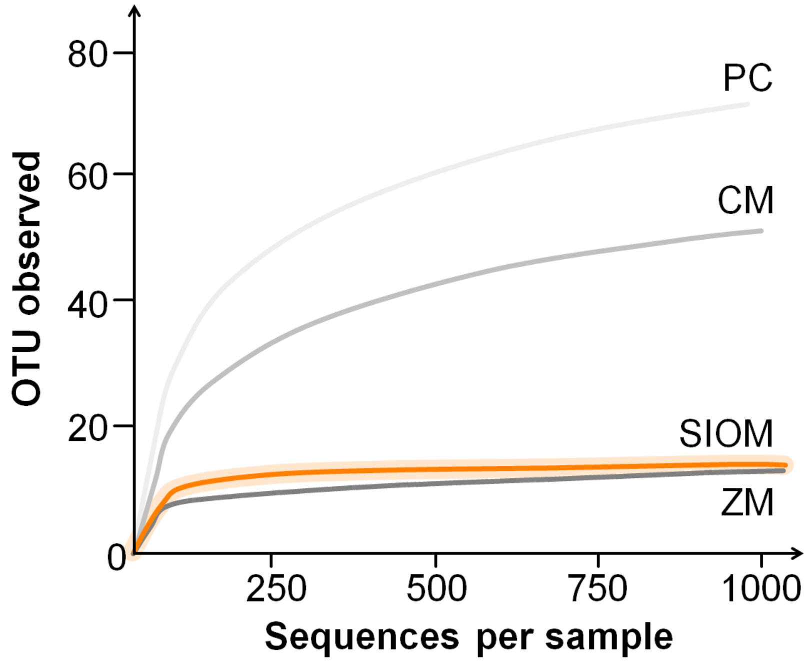

Supplementary Fig. 9. Observed species to sample sequencing rarefaction curve. Operational taxonomic units observed across the sequencing number indicate the sample rarefaction (n=3). PC refers to membrane free incubated salivary sample.

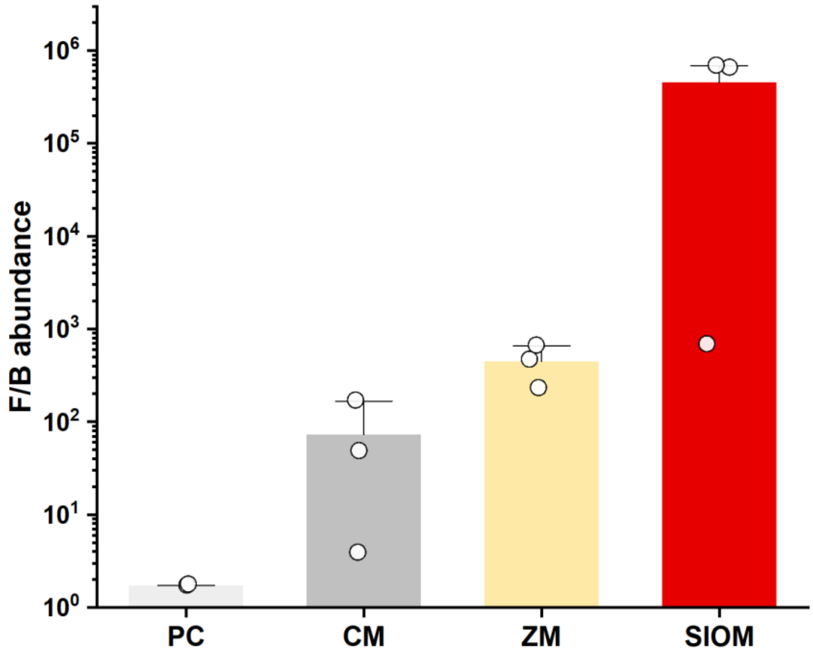

Supplementary Fig. 10. Specific abundance ratio of the positive control (PC; n=2), CM, ZM (n=3) and SIOM (n=3). Multi-fold higher *Firmicutes* to *Bacteroidetes* ratio (F/B) is monitored in *in vitro* human salivary 48 h co-cultured with the ZM and SIOM. The data are presented in mean ± SD. Source data are provided as a Source Data file.

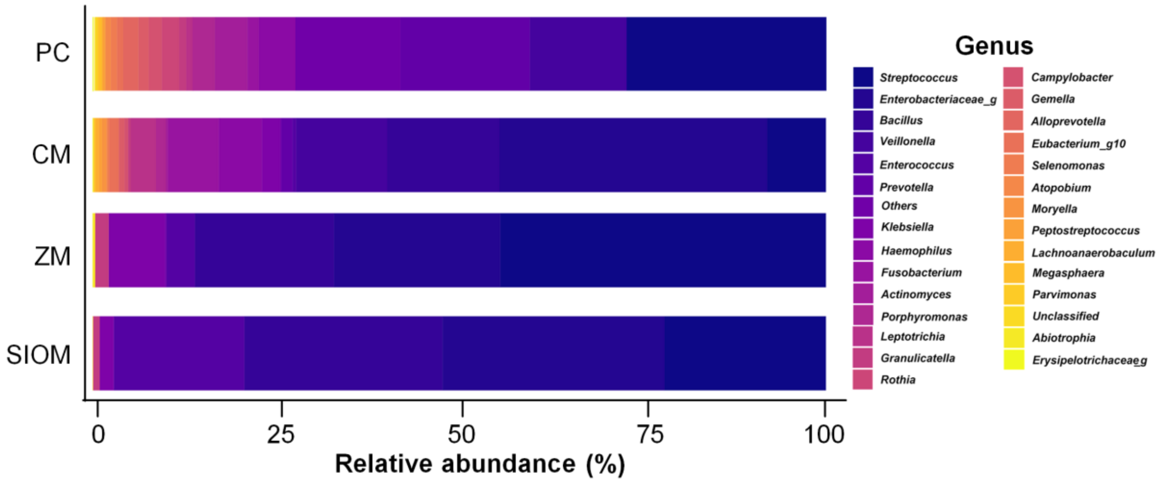

Supplementary Fig. 11. Taxonomic composition for the CM, ZM, and SIOM at the genus-level resolutions in comparison to pooled saliva culture sequences (n=3). PC refers to membrane free incubated salivary sample (n=2).

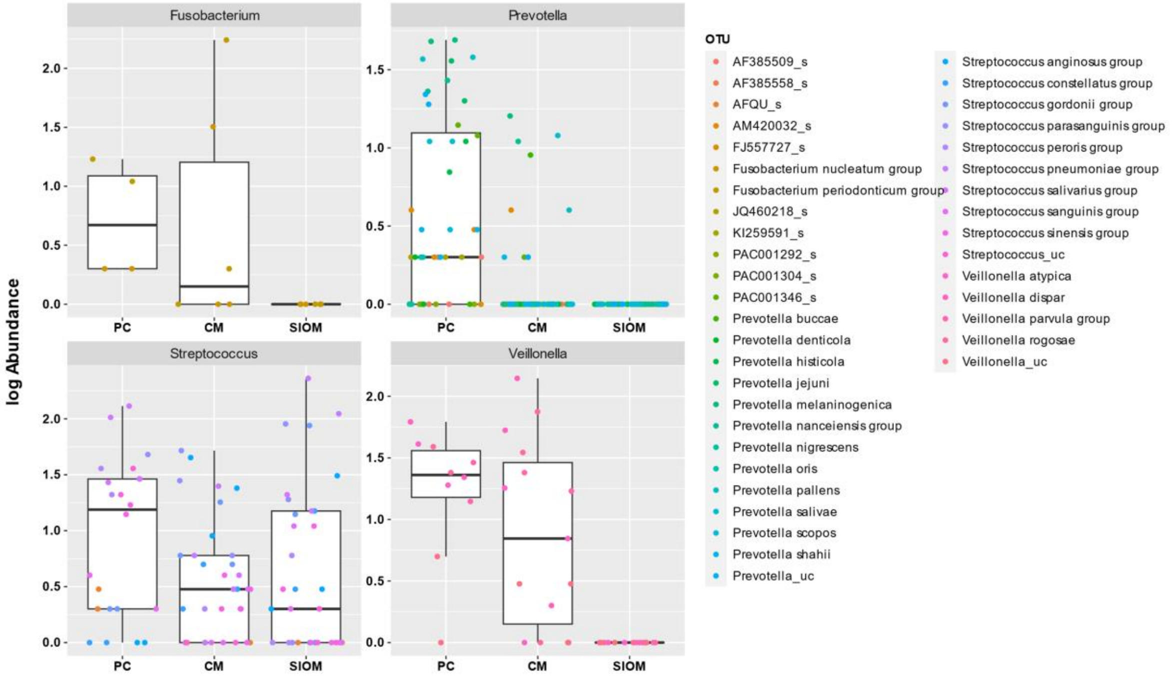

Supplementary Fig. 12. Species level comparison of CM (n=3) and SIOM (n=3). Log abundance distribution pattern observed for the *Fusobacterium*, *Prevotella*, *Streptococcus,* and *Veillonella* genera. PC represents the microbiota expression from pooled salivary incubate without interaction in the absence of occlusive membranes. PC refers to membrane free incubated salivary sample (n=2).

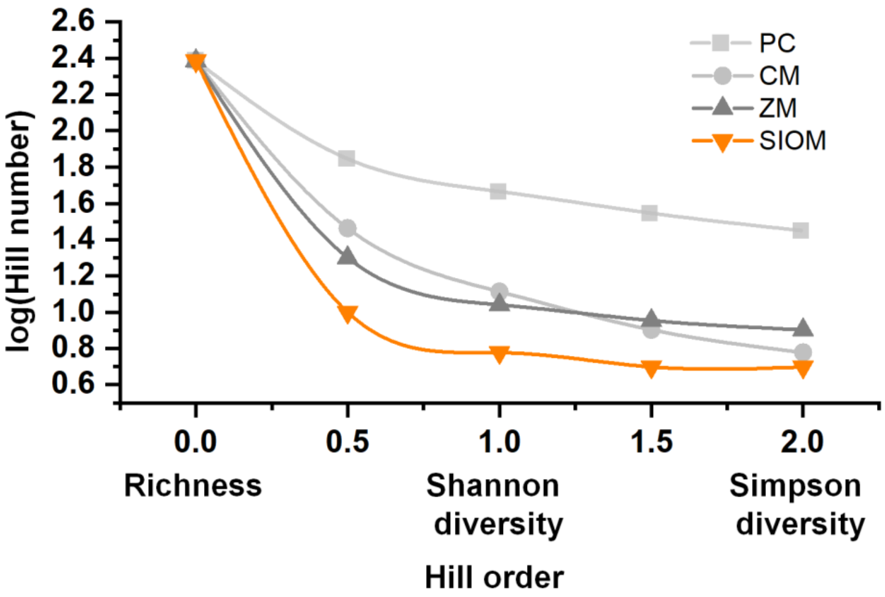

Supplementary Fig. 13. Hill number analysis for the effective number of equally abundant species. True lack of diversity expressed in the ZM and SIOM is acquired through the 16S rRNA sequencing of *in vitro* co-cultured multi-species human salivary microbiota (n=3). PC refers to membrane free incubated salivary sample (n=2). Source data are provided as a Source Data file.

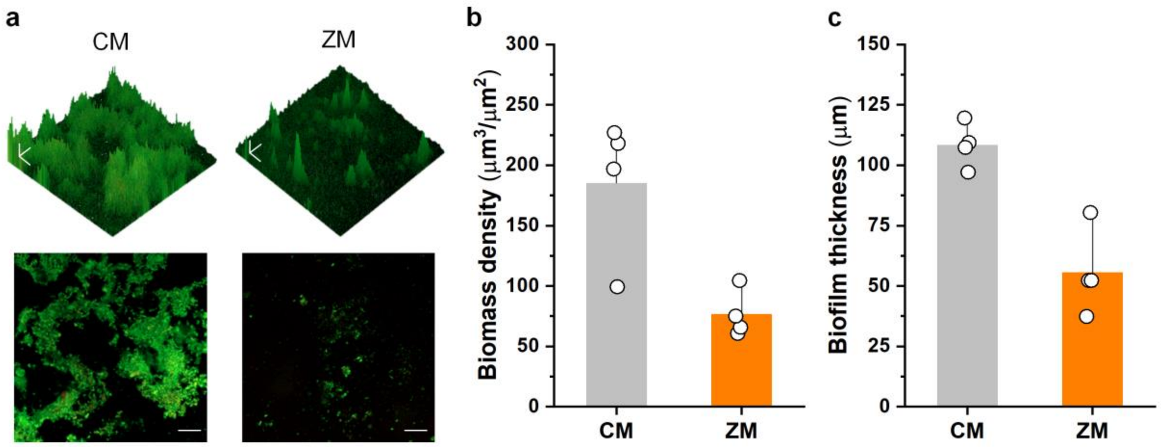

Supplementary Fig. 14. Antifouling property of ZM. a Image of *in vitro* human salivary biofilm (n=4). The green indicates the live bacterial biofilm after 48 h co-cultured with the CM or ZM. Scale bar = 100 μm. **b** Biomass density (n=4; mean ± SD) and **c** biofilm thickness (n=4; mean ± SD) indicate that ZM exhibited approximately 50% suppression of biofilm growth compared to CM.[11] Source data are provided as a Source Data file.

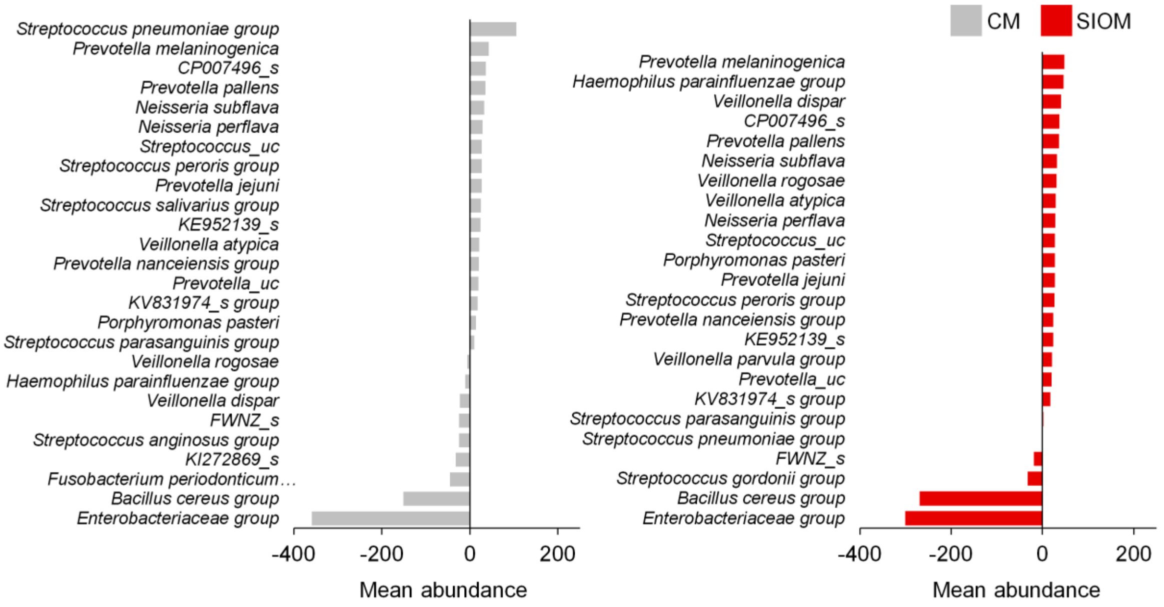

Supplementary Fig. 15. The differences in abundances of taxonomic groups with greater than 1% contribution to dissimilarity to PC. The negative values in *the x*-axis indicate lower abundance in PC compared to CM/SIOM and vice-versa. PC refers to membrane free incubated salivary sample (n=2). n=3 for CM and SIOM.

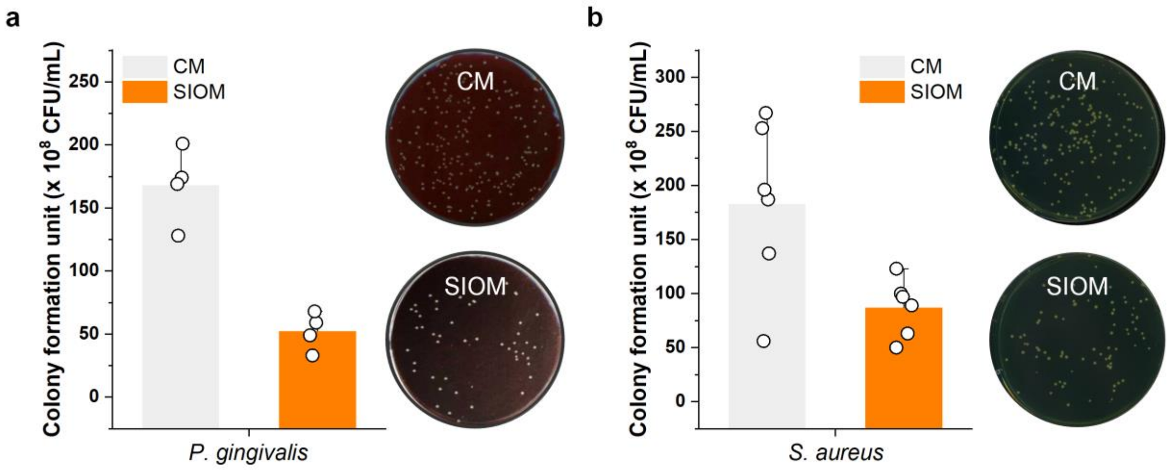

Supplementary Fig. 16. Colony formation unit of **a** *Porphyromonas gingivalis* (n=4; mean ± SD), **b** *Staphylococcus aureus* (n=6; mean ± SD) co-cultured with CM and SIOM. Source data are provided as a Source Data file.

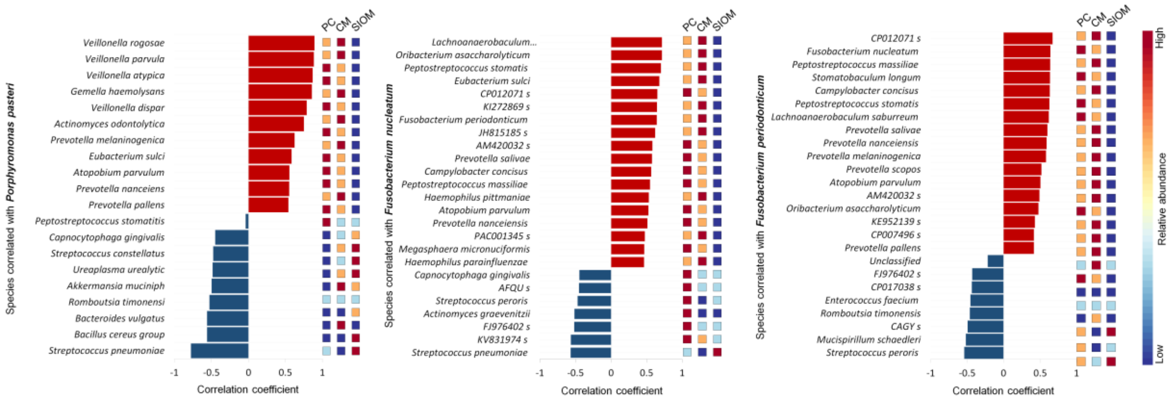

Supplementary Fig. 17. The correlation patterns between *Porphyromonas pasteri*, *Fusobacterium nucleatum*, and *Fusobacterium periodonticum* were analyzed using the Spar CC method (n=3). PC refers to membrane free incubated salivary sample. [12] This statistical approach helps assess the associations between different taxa in microbiome datasets, mainly when dealing with compositional data, and was performed using a web-based platform.[13]

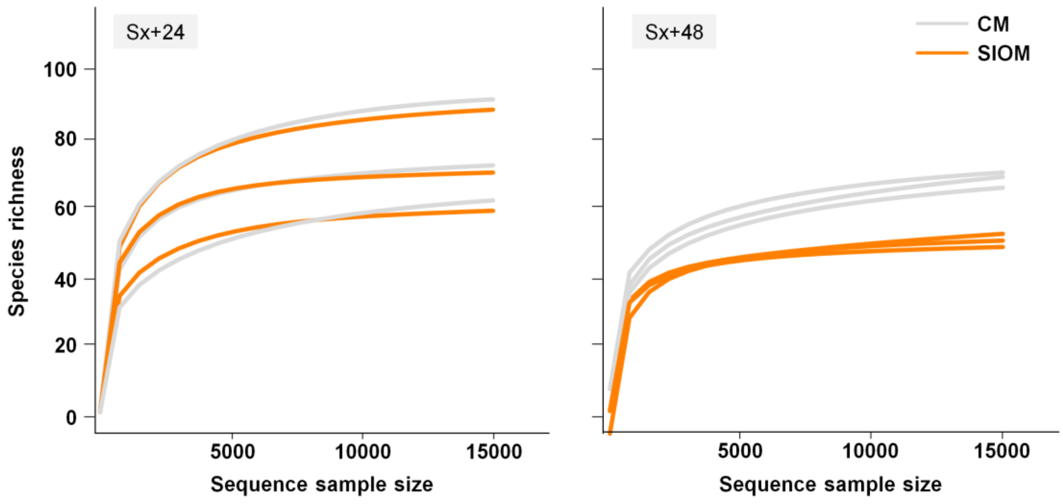

Supplementary Fig. 18. Species richness rarefaction curve. The species richness identified with increasing sample sequence is plotted for *in vivo* samples collected 24 and 48 hours after surgical treatment (Sx). Sx+48 profile shows a distinct separation between the two groups (n=3), with relatively early plateauing observed for SIOM.

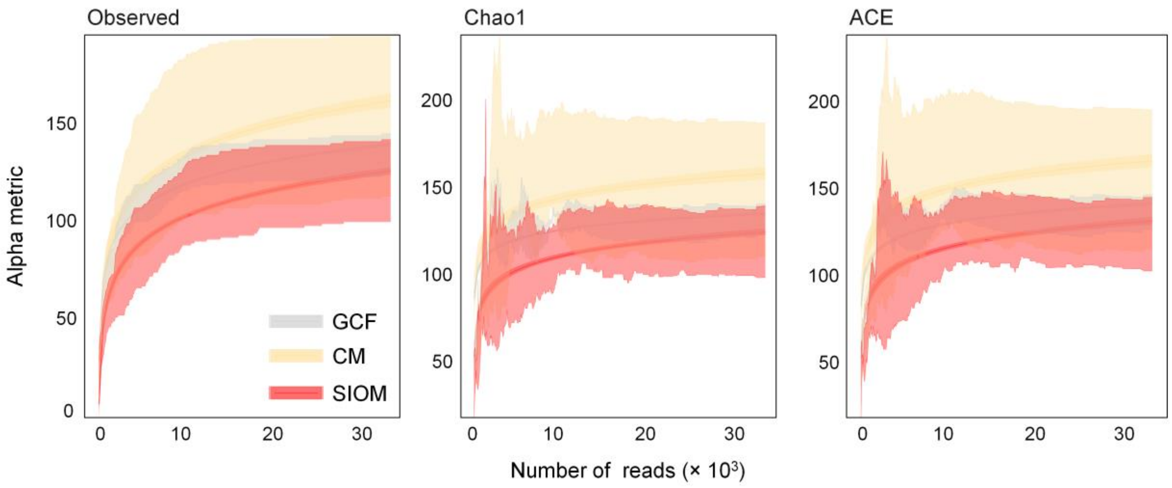

Supplementary Fig. 19. Reduction in the alpha diversity at the level of amplicon sequence variants. The observed diversity provides a direct count of species in a sample. Chao1 and Abundance-based Coverage Estimator (ACE) diversity indices estimate the total species richness, considering both observed and unobserved species.

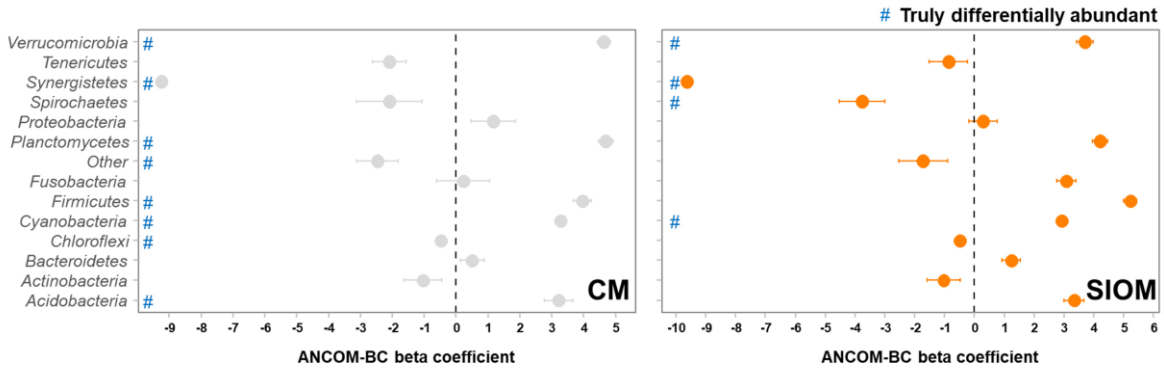

Supplementary Fig. 20. Analysis of the identified taxa from *in vivo* canine model. Differentially abundant taxa identified by ANCOM-BC with co-variate adjustment. Pre-treatment gingival crevicular fluid samples serve as the baseline reference.

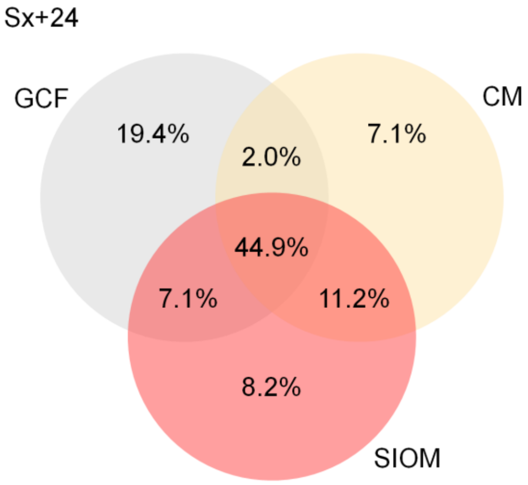

Supplementary Fig. 21. Core-microbiota distribution between the GCF, CM, and SIOM, observed at different time points.

Supplementary Table 1. Quantitative biocompatibility analysis showing response distance from test material interface (scale: cm).

No.	SIOM	[a] HDPE (NC)	[b] ZDEC (PC)
1	0	0	0.51
2	0	0	0.5
3	0	0	0.52
Mean ± SD	0.00 ± 0.00	0.00 ± 0.00	0.51 ± 0.01

[a] HDPE; High Density Polyethylene Film; Negative control (NC)

[b] ZDEC; 0.1% ZDEC polyurethane film; Positive control (PC)

Supplementary Table 2. Qualitative biocompatibility analysis: Reactivity grade

No.	SIOM	[a] HDPE (NC)	[b] ZDEC (PC)
1	1 (Slight)	0 (None)	3 (Moderate)
2	1 (Slight)	0 (None)	3 (Moderate)
3	1 (Slight)	0 (None)	3 (Moderate)
Average	1 (Slight)	0 (None)	3 (Moderate)

[a] HDPE; High Density Polyethylene Film; Negative control (NC)

[b] ZDEC; 0.1% ZDEC polyurethane film; Positive control (PC)

Supplementary Table 3. FDR (false discovery rate) bias-corrected analysis of differentially abundant taxa with analysis of compositions of microbiomes with bias correction (ANCOM-BC)

Phyla	Beta-coefficient (*W*)		Standard error		Differentially abundant		Relative abundance		FDR value (*q*)	
	CM	SIOM	CM	SIOM	CM	SIOM	CM	SIOM	CM	SIOM
Tenericutes	-2.08695	-0.86879184	0.530866	0.63809377	FALSE	FALSE	3.33	1.33	5.74E-02	4.49E-01
Spirochaetes	-2.090111	-3.75868513	1.02842	0.76510948	FALSE	TRUE	197.33	44.00	5.74E-02	5.86E-04*
Proteobacteria	1.160967	0.28546156	0.695246	0.47726797	FALSE	FALSE	1374.00	852.00	3.44E-01	7.75E-01
Fusobacteria	0.220584	3.07034844	0.834404	0.32180151	FALSE	TRUE	9678.67	10601.00	8.25E-01	4.28E-03*
Bacteroidetes	0.500062	1.2329256	0.374642	0.31016438	FALSE	FALSE	10836.67	10717.33	6.81E-01	3.05E-01
Acidobacteria	3.215367	3.33564011	0.448713	0.327173	TRUE	TRUE	108.33	132.67	3.04E-03*	1.99E-02
Verrucomicrobia	4.633183	3.707471	0.21341	0.27674468	TRUE	TRUE	0.00	0.00	1.68E-05*	5.86E-04*
Synergistetes	-9.247514	-9.62984912	0	0	TRUE	TRUE	0.00	0.33	0.00E+00*	0.00E+00*
Planctomycetes	4.692319	4.20788622	0.231824	0.26213618	TRUE	TRUE	0.33	0.33	1.68E-05*	1.20E-04*
Other	-2.463879	-1.71939438	0.658528	0.8269246	TRUE	FALSE	14.33	176.33	2.75E-02*	1.33E-01
Firmicutes	3.963607	5.21980338	0.283852	0.22013747	TRUE	TRUE	11653.00	10296.00	2.58E-04*	1.25E-06*
Cyanobacteria	3.278702	2.91790054	0	0	TRUE	TRUE	1.67	2.67	0.00E+00*	0.00E+00*
Chloroflexi	-0.47846	-0.47881451	0	0	TRUE	TRUE	0.00	0.00	0.00E+00*	0.00E+00*
Actinobacteria	-1.041663	-1.02959918	0.588714	0.56279316	FALSE	FALSE	108.33	132.67	3.79E-01	3.86E-01

#ANCOMBC2 identifies taxa that exhibit significant differences in abundance between groups. The beta coefficient (*W*), is a measure of the effect size representing the change in the relative abundance of a taxon between the groups being compared. It provides an estimate of the magnitude and direction of change in abundance associated with the group. The magnitude and direction of *W* indicate the degree of differential abundance. Larger absolute values of *W* suggest stronger differential abundance, while values closer to zero indicate less pronounced differences in abundance between groups. FDR (*q*) values present the adjusted *p* values by Benjamini & Hochberg correction of false discovery rate. The significance threshold was set at $q < 0.05$. The asterisk symbol in *q* values represents the truly differentially abundant Phyla.

Supplementary Table 4. Information on primers for qPCR analysis

Target Gene	Primer sequence (5' - 3')	
BMP2	Forward	AGCGAGTTCGAGTTGCGGCT
	Reverse	AGCTGCGCACAGTGTTGGCT
OCN	Forward	CCCAGGCGCTACCTGTATCAA
	Reverse	GGTCAGCCAACTCGTCACAGTC
OPN	Forward	ACACATATGATGGCCGAGGTGA
	Reverse	TGTGAGGTGATGTCCTCGTCTGTAG
RUNX2	Forward	AACCCTTAATTTGCACTGGGTCA
	Reverse	CAAATTCCAGCAATGTTTGTGCTAC

References

1. Socransky, S.S., Haffajee, A.D., Cugini, M.A., Smith, C. & Kent, R.L., Jr. Microbial complexes in subgingival plaque. *J. Clin. Periodontol.* **25**, 134-144 (1998).
2. Haffajee, A.D., Socransky, S.S., Patel, M.R. & Song, X. Microbial complexes in supragingival plaque. *Oral Microbiol. Immunol.* **23**, 196-205 (2008).
3. Socransky, S.S. *et al.* Use of checkerboard DNA-DNA hybridization to study complex microbial ecosystems. *Oral Microbiol. Immunol.* **19**, 352-362 (2004).
4. Yu, Y. *et al.* Multifunctional "hydrogel skins" on diverse polymers with arbitrary shapes. *Adv. Mater.* **31**, 1807101 (2019).
5. Nonoyama, T. *et al.* Double-network hydrogels strongly bondable to bones by spontaneous osteogenesis penetration. *Adv. Mater.* **28**, 6740-6745 (2016).
6. De Yoreo, J.J. *et al.* Crystallization by particle attachment in synthetic, biogenic, and geologic environments. *Science* **349**, aaa6760 (2015).
7. Turecek, F. N Cα Bond Dissociation Energies and Kinetics in Amide and Peptide Radicals. Is the Dissociation a Non-ergodic Process? *J. Am. Chem. Soc.* **125**, 5954-5963 (2003).
8. Jiang, W. *et al.* The effects of hydroxyapatite coatings on stress distribution near the dental implant–bone interface. *Appl. Surf. Sci.* **255**, 273-275 (2008).
9. Li, Z. *et al.* A tough hydrogel–hydroxyapatite bone-like composite fabricated in situ by the electrophoresis approach. *J. Mater. Chem. B* **1**, 1755-1764 (2013).
10. Choi, W. *et al.* Regulation of the Inevitable Water-Responsivity of Silk Fibroin Biopolymer by Polar Amino Acid Activation. *ACS Nano* **16**, 17274-17288 (2022).
11. Choi, W. *et al.* Reverse actuation of polyelectrolyte effect for in vivo antifouling. *ACS Nano* **15**, 6811-6828 (2021).

12. Friedman, J. & Alm, E.J. Inferring Correlation Networks from Genomic Survey Data. *PLoS Comput. Biol.* **8**, e1002687 (2012).

13. Chong, J., Liu, P., Zhou, G. & Xia, J. Using MicrobiomeAnalyst for comprehensive statistical, functional, and meta-analysis of microbiome data. *Nat. Protoc.* **15**, 799-821 (2020).
