# Supplementary material for: Occlusive membranes for guided regeneration of inflamed tissue defects
Source: Nat Commun. 2023 Nov 24;14:7687. doi: 10.1038/s41467-023-43428-3 (PMC10673922; doi:10.1038/s41467-023-43428-3)
Supplement: Supplementary file 3 — Description of additional supplementary files [file 41467_2023_43428_MOESM3_ESM.pdf]

Description of additional supplementary files

Supplementary Data 1. Core taxonomic groups identified from downstream analysis of in vitro co-culture of the human salivary microbiome
