# Supplementary material for: Occlusive membranes for guided regeneration of inflamed tissue defects
Source: Nat Commun. 2023 Nov 24;14:7687. doi: 10.1038/s41467-023-43428-3 (PMC10673922; doi:10.1038/s41467-023-43428-3)
Supplement: Supplementary file 5 — Reporting Summary [file 41467_2023_43428_MOESM5_ESM.pdf]

nature portfolio

Corresponding author(s): Jinkee Hong
Last updated by author(s): Oct 23, 2023

Reporting Summary

Nature Portfolio wishes to improve the reproducibility of the work that we publish. This form provides structure for consistency and transparency in reporting. For further information on Nature Portfolio policies, see our Editorial Policies and the Editorial Policy Checklist.

Statistics

For all statistical analyses, confirm that the following items are present in the figure legend, table legend, main text, or Methods section.

n/a	Confirmed	
☐	☒	The exact sample size (*n*) for each experimental group/condition, given as a discrete number and unit of measurement
☐	☒	A statement on whether measurements were taken from distinct samples or whether the same sample was measured repeatedly
☐	☒	The statistical test(s) used AND whether they are one- or two-sided<br>*Only common tests should be described solely by name; describe more complex techniques in the Methods section.*
☐	☒	A description of all covariates tested
☐	☒	A description of any assumptions or corrections, such as tests of normality and adjustment for multiple comparisons
☐	☒	A full description of the statistical parameters including central tendency (e.g. means) or other basic estimates (e.g. regression coefficient) AND variation (e.g. standard deviation) or associated estimates of uncertainty (e.g. confidence intervals)
☐	☒	For null hypothesis testing, the test statistic (e.g. *F*, *t*, *r*) with confidence intervals, effect sizes, degrees of freedom and *P* value noted<br>*Give P values as exact values whenever suitable.*
☒	☐	For Bayesian analysis, information on the choice of priors and Markov chain Monte Carlo settings
☒	☐	For hierarchical and complex designs, identification of the appropriate level for tests and full reporting of outcomes
☒	☐	Estimates of effect sizes (e.g. Cohen's *d*, Pearson's *r*), indicating how they were calculated

Our web collection on statistics for biologists contains articles on many of the points above.

Software and code

Policy information about availability of computer code

Data collection	No software was used
Data analysis	Fiji Image J 1.8.0 was used to perform quantitative analysis of fluorescence intensity from confocal images in in vitro biofilm assay.<br>Paleontological Statistics Software (PAST3; SIMPER) (Version 4.11 for Windows) and R studio (Version 1.4.1106) statistical platforms were utilized to analyze data from genomic sequencing of microbiota.<br>The R packages (version number) used for analysis in the present study are:<br>phyloseq (v1.44.0) R package<br>eulerr (v.6.1.1) R package<br>vegan (v2.5-7) R package<br>mia (v1.8.0) R package<br>phylosmith (v1.0.6) R package<br>ANCOM-BC (v.1.0.1) R package<br>pibble (v1.0.4) R package<br>Hilldiv (v 1.5.1) R package<br>dysbiosisR (v1.0.4) R package (https://github.com/microsud/dysbiosisR)

For manuscripts utilizing custom algorithms or software that are central to the research but not yet described in published literature, software must be made available to editors and reviewers. We strongly encourage code deposition in a community repository (e.g. GitHub). See the Nature Portfolio guidelines for submitting code & software for further information.

Data

Policy information about availability of data

All manuscripts must include a data availability statement. This statement should provide the following information, where applicable:

- Accession codes, unique identifiers, or web links for publicly available datasets
- A description of any restrictions on data availability
- For clinical datasets or third party data, please ensure that the statement adheres to our policy

All data is available in the main text or supplementary materials. The data that support the findings of this study are available from the corresponding authors on reasonable request. Source data are provided as a Source Data File. The raw sequencing data generated in this study data have been submitted to the NCBI BioProject database under accession number PRJNA981675 [https ://www.ncbi.nlm.nih.gov/sra/PRJNA981675].

Research involving human participants, their data, or biological material

Policy information about studies with human participants or human data. See also policy information about sex, gender (identity/presentation), and sexual orientation and race, ethnicity and racism.

Reporting on sex and gender	Human-saliva-derived biofilm in vitro analyses were carried out with saliva from 6-human donors (male=3, female=3). The age and gender of the donor is irrelevant for this study.
Reporting on race, ethnicity, or other socially relevant groupings	The study comprised of human salivary biospecimen where the characteristics of healthy donor individuals (race/ethinicity etc.) was not a variable of comparison and relevant to the study.
Population characteristics	The salivary samples were pooled for analysis and results are not independent of the participant groups or their characteristics.
Recruitment	The participation was voluntary. Based on self-reported information, only the participants that were systematically healthy, had no periodontal disease or active caries and has not taken any antibiotics for at least 3 months, were included as eldigible donors. The salivary samples (2-4 mL) were collected from a total of six healthy donors. The unsimulated saliva was collected in a sterilized laboratory tube, pooled into a single laboratory tube and stored in -80°C before experiment. Self-reported sex or gender of the participants was not considered for saliva sampling. The pooled nature of the sample storage mimiced natural variations within population. No biases that could impact the results were identified.
Ethics oversight	The saliva was obtained following the ethical principles of the 64th World Medical Association Declaration of Helsinki and procedures approved by the institutional review board of the Yonsei University Dental Hospital (Republic of Korea) (2-2019-0049). The consent was obtained from all participants before donating saliva.

Note that full information on the approval of the study protocol must also be provided in the manuscript.

Field-specific reporting

Please select the one below that is the best fit for your research. If you are not sure, read the appropriate sections before making your selection.

[x] Life sciences   [ ] Behavioural & social sciences   [ ] Ecological, evolutionary & environmental sciences

For a reference copy of the document with all sections, see nature.com/documents/nr-reporting-summary-flat.pdf

Life sciences study design

All studies must disclose on these points even when the disclosure is negative.

Sample size	The sample sizes were estimated based on previous studies and have been listed in the manuscript.<br>At least 3 random samples were selected for statistical analysis in each experiment.
Data exclusions	No data were excluded from analyses.
Replication	All in vitro and in vivo results are representative of three to six independents. Successful attempts for replication were observed.
Randomization	All samples were randomly allocated to experimental groups.
Blinding	All data collection and analysis were blinded to group allocation in vitro for biological experiments. Due to differences in handling during experiment, such as additional sterilization steps with experiment group, blinding was not possible for the intra oral split mouth design.

Reporting for specific materials, systems and methods

We require information from authors about some types of materials, experimental systems and methods used in many studies. Here, indicate whether each material, system or method listed is relevant to your study. If you are not sure if a list item applies to your research, read the appropriate section before selecting a response.

Materials & experimental systems

n/a	Involved in the study
☐	☒ Antibodies
☐	☒ Eukaryotic cell lines
☒	☐ Palaeontology and archaeology
☐	☒ Animals and other organisms
☒	☐ Clinical data
☒	☐ Dual use research of concern
☒	☐ Plants

Methods

n/a	Involved in the study
☒	☐ ChIP-seq
☒	☐ Flow cytometry
☒	☐ MRI-based neuroimaging

Antibodies

Antibodies used	MPO; Supplier name: Antibodies; Catalog number: ABIN5013150; Clone name: 1F10; Applicable: IHC; Dilution: 1:200<br>CD86; Supplier name: Antibodies; Catalog number: ABIN736701; Clone name: BU63; Applicable: IHC; Dilution: 1:200<br>CD20; Supplier name: Novus Biologicals; Catalog number: NBP2-70362H; Clone name: OTI4B3; Applicable: IHC; Dilution: 1:200
Validation	All the commercial antibodies have been validated by manufacturers and the statements can be found on the manufacturers' websites. Antibodies were all validated by positive control experiments.

Eukaryotic cell lines

Policy information about cell lines and Sex and Gender in Research

Cell line source(s)	Human Bone Marrow-Derived Mesenchymal Stem Cells; Normal, purchased from ATCC (PCS-500-012)
Authentication	Authentication by visual inspection.
Mycoplasma contamination	The quality control reports confirmed negativity for mycoplasma contamination. No additional tests were performed.
Commonly misidentified lines (See ICLAC register)	No commonly misidentified lines were used in tis study.

Animals and other research organisms

Policy information about studies involving animals; ARRIVE guidelines recommended for reporting animal research, and Sex and Gender in Research

Laboratory animals	6-male mongrel dogs, 10–14 months of age and weighing 25-30 kg were included in the present study.
Wild animals	No wild animals were used in this study.
Reporting on sex	In order to avoid experimental differences caused by animal sex, animals of the same sex were used.
Field-collected samples	No field-collected sample were used in this study.
Ethics oversight	All the animal experiments were approved by the Institutional Animal Care and Use Committee at Yonsei Medical Center, Seoul, Korea (Approval No.2022-0086).

Note that full information on the approval of the study protocol must also be provided in the manuscript.
